# Barriers and facilitators to reducing anticholinergic burden: a qualitative systematic review

**DOI:** 10.1007/s11096-021-01293-4

**Published:** 2021-06-25

**Authors:** Carrie Stewart, Katie Gallacher, Athagran Nakham, Moira Cruickshank, Rumana Newlands, Christine Bond, Phyo Kyaw Myint, Debi Bhattacharya, Frances S. Mair

**Affiliations:** 1 Ageing Clinical and Experimental Research (ACER) Team, Institute of Applied Health Sciences, University of Aberdeen, 1:128, Polwarth Building, Foresterhill Health Campus, Aberdeen, AB25 2ZD UK; 2 General Practice and Primary Care, University of Glasgow, Glasgow, UK; 3 Health Services Research Unit, Institute of Applied Health Sciences, University of Aberdeen, Aberdeen, UK; 4 Primary Care, Institute of Applied Health Sciences, University of Aberdeen, Aberdeen, UK; 5 School of Pharmacy, University of East Anglia, Norwich, UK

**Keywords:** Anticholinergics, Deprescribing, Intervention implementation, Qualitative research, Systematic review

## Abstract

*Background* Despite common use, anticholinergic medications have been associated with serious health risks. Interventions to reduce their use are being developed and there is a need to understand their implementation into clinical care. *Aim of review* This systematic review aims to identify and analyse qualitative research studies exploring the barriers and facilitators to reducing anticholinergic burden. *Methods* Medline (OVID), EMBASE (OVID), CINAHL (EMBSCO) and PsycINFO (OVID) were searched using comprehensive search terms. Peer reviewed studies published in English presenting qualitative research in relation to the barriers and facilitators of deprescribing anticholinergic medications, involving patients, carers or health professionals were eligible. Normalization Process Theory was used to explore and explain the data. *Results* Of 1764 identified studies, two were eligible and both involved healthcare professionals (23 general practitioners, 13 specialist clinicians and 12 pharmacists). No studies were identified that involved patients or carers. Barriers to collaborative working often resulted in poor motivation to reduce anticholinergic use. Low confidence, system resources and organisation of care also hindered anticholinergic burden reduction. Good communication and relationships with patients, carers and other healthcare professionals were reported as important for successful anticholinergic burden reduction. Having a named person for prescribing decisions, and clear role boundaries, were also important facilitators. *Conclusions* This review identified important barriers and facilitators to anticholinergic burden reduction from healthcare provider perspectives which can inform implementation of such deprescribing interventions. Studies exploring patient and carer perspectives are presently absent but are required to ensure person-centeredness and feasibility of future interventions.

**Supplementary Information:**

The online version contains supplementary material available at 10.1007/s11096-021-01293-4.

## Impacts on practice

- Important personal and structural factors such as confidence, time, inter-professional relationships and effective communication can influence engagement with, and success of, anticholinergic burden reduction initiatives and should be considered within the design of such initiatives.
- Given the limited published evidence, we recommend researchers, clinicians and service providers consider evaluating implementation issues when planning future anticholinergic deprescribing interventions.

## Introduction

Anticholinergic drugs, used to treat common conditions including gastrointestinal disorders, overactive bladder, depression, and cardiovascular disease [1–3], have many side effects including dry mouth, constipation, increased heart rate, and confusion [1, 2]. Estimates regarding the number of adults using one or more anticholinergic medications range considerably, from 11 to 47% [4–6]. Concomitant use of multiple medications with anticholinergic properties has a cumulative effect, known an anticholinergic burden (ACB) [2, 7]. Greater ACB has been associated with impaired physical and cognitive function, falls, cardiovascular events and mortality [8–13]. Much of the present evidence behind these adverse events was determined from older persons; however, recent evidence suggests use of anticholinergics in mid-life may pose similar risks in later life [13], especially its impact on cognitive impairment [14]. Given these significant risks, several interventions aimed at reducing ACB have been developed and tested. Our recent systematic review identified eight such studies (PROSPERO registration CRD42018089764) [15]. Interventions varied widely regarding their design and setting (e.g. community, nursing homes, acute care), person delivering the interventions (e.g. pharmacist, pharmacologist, physician), and how recommendations were identified and made (e.g. face to face, over the telephone or virtually) [15]. Seven of the eight studies reported positive improvements regarding ACB [15]. However, little is known about the implementation of such interventions, for example what factors can increase or decrease successful embedding and integration of them into practice.

The literature surrounding the broader concept of ‘deprescribing’ reveals common barriers and facilitators [16–19]. These include concerns about negative consequences arising from stopping medications, and a lack of ongoing support [16–19]. Conversely, patient motivation, support for the prescriber and patient, and prescriber and patient beliefs, all support the deprescribing process [16–19]. However, it is unknown if the barriers and facilitators towards reducing ACB differ from those for general deprescribing of inappropriate medications. Anticholinergic drugs and medical conditions associated with their use may present unique challenges. Understanding this is essential to inform the development and design of ACB reduction interventions, in line with the Medical Research Council (MRC) Complex Intervention Framework [20]. Addressing these factors will increase the chances of successfully implementing interventions into practice [20] A robust theoretical framework to underpin our data analysis is also required to help us move from a descriptive account to one that is more explanatory, which is advocated when developing complex interventions [20]. NPT is a well-developed theory for understanding the factors involved in successful implementation [21–25]. NPT consists of four constructs: coherence, which addresses the sense-making work that people participating in an intervention have to undertake; cognitive participation, the engagement work that is undertaken by or between participants; collective action, the operational work and tasks that people have to do to enact the intervention; and reflexive monitoring, the appraisal work people undertake in relation to the intervention [24].

### Aim of review

This study aims to identify facilitators and barriers to the implementation of interventions to reduce ACB amongst adult patients from the perspectives of healthcare professionals, patients and carers from the available qualitative evidence and through the lens of NPT. This review will seek to identify key research gaps where future implementation research is required.

### Methods

The systematic review protocol is PROSPERO registered (CRD42018109084), published [26], conducted in line with the general principles of the Cochrane Handbook for Systematic Reviews of Interventions [27], ENTREQ (Enhancing Transparency in Reporting the Synthesis of Qualitative Research) [28] and reported in accordance with the PRISMA statement [29]. See Supplementary file 1 for further protocol detail and PRISMA checklist.

### Search strategy and selection criteria

Four electronic databases were searched: Medline (OVID), EMBASE (OVID), CINAHL (EMBSCO) and PsycINFO (OVID). The search strategy developed for Ovid MEDLINE was adapted for the other databases (presented in Supplementary material 1). Bibliographies and citations of included publications were searched manually for eligible studies. Inclusion/exclusion criteria were determined using a modified PICO (population, intervention, control and outcome) framework [30], presented in Table 1. Language exclusions were conducted by hand. No date restrictions were imposed.

**Table 1:** Study eligibility criteria

	Inclusion criteria	Exclusion criteria
Population (Participants)	Persons aged ≥ 18 years of age Persons using one or more anticholinergic medications Carer/proxy (e.g. a person answering on behalf of the patient) for an adult using one or more anticholinergic medications Healthcare professional (e.g. physicians, nurses, pharmacists) involved in the care of adults using one or more anticholinergic medications	Persons aged < 18 years
Setting	Primary care Community Nursing home Outpatient clinics Day hospitals/centres/care facilities Rehabilitation services	Acute care/ inpatients Palliative care
Intervention	Original research findings examining attitudes to deprescribing/medication switching in relation to anticholinergic medication	
Study type/design	Qualitative research (face-to-face or telephone approaches) Full papers published in peer-reviewed journals Published in English	Quantitative research
Controls	None	
Outcome	Barriers and facilitators to deprescribing or medication switching in relation to anticholinergic medications	

### Data collection and extraction

Searches were conducted on 2nd November 2018 and updated on the 31st March 2020. Identified studies were transferred into RefWorks (ProQuest LLC) [31] and then into Covidence (Veritas Health Innovation Ltd) [32] for screening. Two independent reviewers (shared between CS, KG, AN, MC, RN, CB, PKM, DB) screened each article title, abstract and full text using the eligibility criteria. Study authors were contacted where full-texts could not be found. Discrepancies between two reviewers were resolved by a third reviewer (FSM).

A standardised data extraction form was developed. Study descriptives such as publication year, author, location, setting etc. were collected alongside relevant qualitative data and information required to ascertain quality. Two reviewers (CS, KG) completed data extraction. Disagreements were discussed and resolved within the wider research team.

### Quality assessment

Two reviewers (CS, KG) independently assessed risk of bias using the Critical Appraisal Skills Programme qualitative checklist (2018) checklist [33]. Quality assessments were used to describe reliability and validity of the body of evidence.

### Analysis

Two reviewers (CS, KG) conducted data analysis with the wider research team available for arbitration. Qualitative data were exported into Microsoft Excel [34] to facilitate data analysis. Normalization Process Theory (NPT) served as the underpinning conceptual framework. A coding sheet underpinned by NPT was developed to provide a framework for categorising the data (see Supplementary file 1). This was adapted and refined during analysis and a note was taken of any data that fell outside of the framework.

## Results

**Fig. 1:**
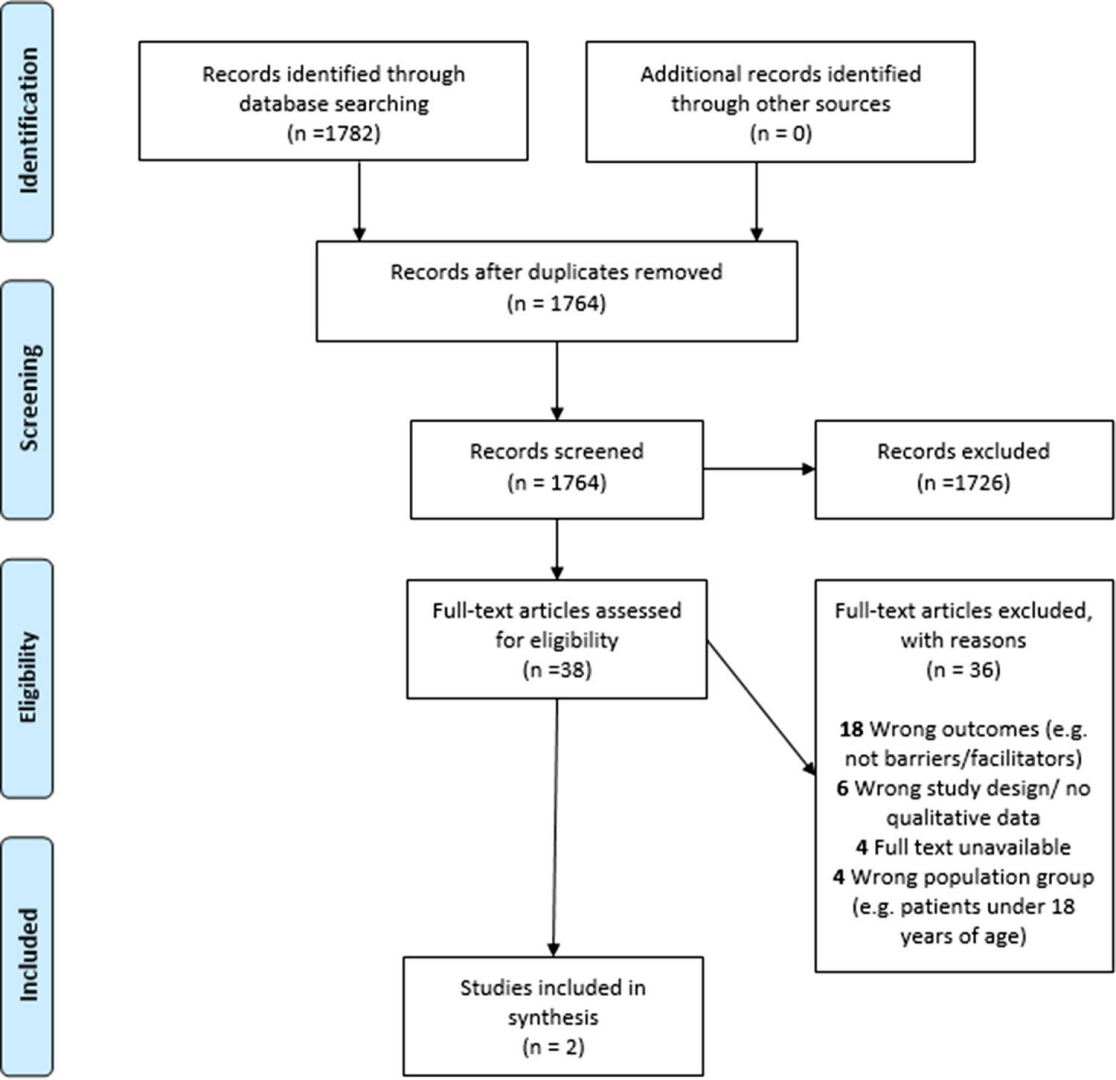
PRISMA flowchart

Identified studies were transferred to Covidence and after deduplication, 1764 studies remained. Following title and abstract screening, 38 articles were reviewed by full text and two articles met our inclusion criteria (exclusion reasons shown in PRISMA Flowchart, Fig. 1). Both studies [35, 36] were conducted in Australia and involved 48 healthcare professionals (23 General Practitioners (GP), 13 specialist clinicians (SP) and 12 accredited pharmacists (AP)). One study was exclusive to primary care [35] while the other spanned primary, community, and secondary care settings [36]. Both studies specifically targeted older people [35, 36]. No studies included patients or carers as participants. The studies differed in their context. Gnjidic et al. [35] obtained feedback from GPs who had participated in an ACB reduction intervention. The study by Kouladjian et al. [36] asked about implementation hypothetically (participants had not been involved in an intervention). In terms of design, the study reported by Gnjidic et al. [35] was a cluster randomized controlled trial and the qualitative data were obtained from GPs’ feedback, obtained through written or verbal communication. In the study by Kouladjian et al. [36] qualitative data were obtained through semi-structured interviews and focus groups. CASP assessment found that both studies reported clear aims and recruitment strategies and tackled a research question appropriate for qualitative research. However, several methodological concerns were evident (see Table 2). Both studies reported limited detail on data collection and analysis [35, 36].

**Table 2:** Critical Appraisal Skills Program (CASP) assessment of included studies (n = 2)^a^

Reviewer	Paper	Clear aim	Approp. for qualitative research	Approp. design	Approp. recruitment strategy	Adequate data collection	Researcher/participant relationship	Ethics considered	Rigorous analysis	Clear findings	Valuable research
Reviewer 1	Kouladjian et al. [36]	Y	Y	Y	Y	Y	U	Y	U	Y	Y
Reviewer 2	Kouladjian et al. [36]	Y	Y	Y	Y	Y	N	Y	U	Y	Y
Reviewer 1	Gnjidic et al. [35]	U	Y	U	Y	U	N	Y	N	N	U
Reviewer 2	Gnjidic et al. [35]	N	Y	U	Y	U	N	Y	N	N	U

*Approp.* Appropriate, *Y* yes, *N* no, *U* unclear

^a^No third reviewer arbitration was required

**Fig. 2:**
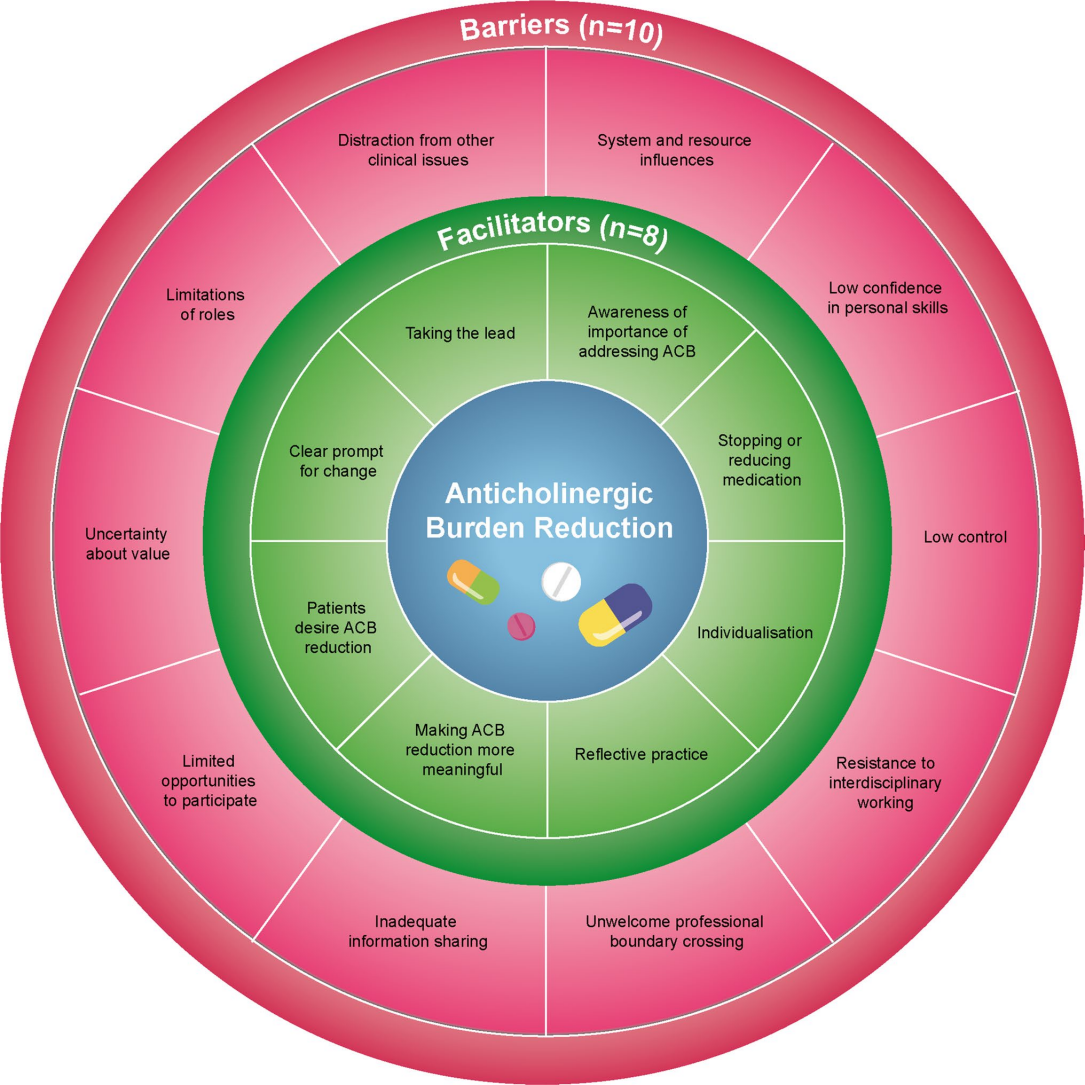
An illustration of barriers and facilitators of ACB reduction from the perspective of healthcare professionals

Across the four NPT constructs we identified data that fell within 8 core themes and 19 sub-themes (illustrated in Table 3). No relevant data fell outside the NPT framework. Figure 2 presents the identified barriers and facilitators for illustrative purposes.

**Table 3:** Overview of identified NPT themes, facilitators and barriers

NPT construct	Core theme	Facilitators	Barriers
Coherence (Sense making)	Differentiation	Clear prompt for change	Distraction from other clinical issues
	Tasks	Stopping or reducing medication Taking the lead Individualisation	Limitations of roles
	Value	Awareness of importance of addressing ACB Patients desire ACB reduction	Uncertainty about value
Cognitive participation (Engagement)	Enrolment		Perceived lack of control Inadequate information sharing Resistance to interdisciplinary working Unwelcome professional boundary crossing Limited opportunities to participate
Collective action (Operationalising ACB reduction)	Skills		Low confidence in personal skills
	Contextual		System and resource influences
Reflexive monitoring (Monitoring and appraising)	Reconfiguration	Making ACB reduction more meaningful	
	Appraisal	Reflective practice

### Coherence (Sense making)

This theme describes how participants make sense of and plan ACB reduction. The data depict what participants envision ACB reduction to look like (differentiation), how it is valued and what tasks it involves. We identified six facilitators of ACB reduction; having a clear prompt for change, having someone take the lead, making it individualised, able to stop or reduce medications, being aware of the importance of ACB reduction, and believing patients desire ACB reduction. We also identified 3 barriers to ACB reduction; distraction from other clinical issues, limitations of roles (professional) and uncertainty about the value of ACB reduction.

There were several statements made in reference to how ACB reduction interventions acted as a *clear prompt for change*; an alert system of possible problems that was seen by some to be a useful approach to bringing potential problems to the forefront [36]. Converse to this, there were concerns from some that an intervention focusing upon ACB may be a *distraction from other clinical issues*, such as other harmful medications without anticholinergic properties [36]. This appeared related to concerns about embedding ACB reduction interventions alongside clinical judgement about other aspects of care.

Many statements reflected a general understanding that ACB reduction involved *stopping or reducing medication* with anticholinergic properties through identifying problem medications or side effects [35, 36]. This task may be undertaken by the individual (prescriber) or involve making recommendations to prescribers [36]. Many accounts reported the need for someone to be *taking the lead* in facilitating this, usually through collaborative communication with patients, family, carers, and other health professionals [36]. This leadership role required facilitating discussions, and negotiation, principally with patients and other health professionals [36]. However, there were uncertainties as to who should lead in these conversations. An important aspect of ACB reduction indicated by several healthcare professionals is the need for *individualisation,* making it personalised for patients [36], weighing up an individual’s risks and benefits to reach the best recommendations for that patient [36].

Perceived *limitations of their role* was expressed as a barrier to supporting ACB reduction by participants. For some, particularly pharmacists, limitations came from recommendations not being followed by prescribers, leading to participants questioning the value of these attempts at ACB reduction [36]. For others, limitations in what they could achieve stemmed from a conflict in priorities between patient-centred care and the needs and wishes of the patient’s carer [36].

Participants demonstrated *awareness of the importance of ACB reduction* and understood the risks of anticholinergic medications and why their use should be reviewed [35, 36]. Two participants [36] perceived that ACB reduction was *desired by patients*, particularly older patients. However, there were also some *uncertainties about the value of ACB reduction*, often presented in relation to the need for individualisation [35, 36]; it was perceived by some that ACB reduction was not necessarily an achievable goal for all patients. Questioning the value was also framed through the participants’ lack of knowledge or confidence in relation to tackling perceived difficulties, such as long-term use of benzodiazepines.

### Cognitive participation (Engagement)

This theme describes the relationship work involved in ACB reduction (enrolment), the qualities or characteristics of that work, who is involved, and relationship difficulties faced. We identified five barriers to ACB reduction; perceived lack of control, inadequate information sharing, resistance towards interdisciplinary working, unwelcome professional boundary crossing, and limited opportunities to participate in ACB reduction tasks.

The sense of *low control* over ACB reduction from participants described earlier [36] was sometimes linked to poor inter-professional relationships and a barrier to engaging with relevant others to perform necessary tasks for ACB reduction. However, low control arising from structural or organisational issues were also cited. For example, the involvement of multiple providers made it difficult to control what a patient was being prescribed. This resonates with statements made referencing *inadequate information sharing*, which impacted on ability to fully understand patients ACB and act upon it [36].

Several references were made to there being *resistance to inter-disciplinary working* between health professionals [35, 36]. Sometimes resistance was expressed in terms of professional conflict regarding clinical opinions about the necessity for particular medications [36]. Many statements suggest concerns about *unwelcome professional boundary crossing* when several health professionals are involved in a patient’s care [35, 36]. It was perceived that recommendations made by pharmacists were not always actioned by the GP. There were also concerns from GPs about changing medications prescribed by specialists. There was also confusion about the roles of different health professionals in regard to ACB reduction. Conflict may arise due to poor communication between health professionals, because there are often very limited opportunities to participate in multidisciplinary discussions regarding a patient’s medications [36]. This could be frustrating, leaving one party feeling aggrieved that their perspective has not been considered.

### Collective action (Operationalising ACB reduction)

This theme describes the resources perceived to be needed for ACB reduction, organisational and environmental influences, and impact on workload. We identified two barriers to ACB reduction; low confidence in personal skills and system and resource influences.

Several participants raised concerns about how *system and resource* issues influence ACB reduction success [36]. These ranged from a lack of time in consultations, to health insurance systems which actively make it more difficult to reduce prescribing of some medications. The way health care systems operate was also raised as an additional challenge for those conducting reviews remotely from the prescriber [36]. Several statements reported how participants were becoming frustrated at the lack of recommendations being upheld [36]. Participants across both studies reported *low confidence in personal skills* to tackle ACB and uncertainties about how to address it were at times linked with doubts that it may not be the correct thing to do [35, 36].

### Reflexive monitoring (Monitoring and appraising)

This theme describes the monitoring of patients before and after ACB reduction, and how the value of ACB reduction is determined through *reflective practice*. We identified two facilitators of ACB reduction; making ACB meaningful and reflective practice.

There were statements regarding the repetitive and iterative nature of ACB reviews [36]. Participants reported how ACB reduction was trialled and then reviewed to evaluate the impact of any changes made [36]. It was this follow-up that allowed participants to evaluate how effective their recommendations had been, suggestive of *reflective practice*. Furthering the idea of *reflective practice*, several suggestions were made [36] regarding how *making ACB reduction more meaningful* could enhance ACB reduction. For example, some participants felt a numerical score would be easier to understand and engage patients with and would provide something measurable to evaluate success against.

## Discussion

To the best of our knowledge, this is the first study to systematically review qualitative studies exploring the barriers and facilitators to ACB reduction. We identified two eligible studies [35, 36] which involved healthcare professionals only. Participants varied in their beliefs in relation to the need and value of ACB reduction, particularly in relation to its importance in comparison to other clinical issues. Difficulties in collaborative working often resulted in perceived low control and poor motivation. Concerns about personal knowledge and confidence, system resources and organisation of care were also raised as barriers. Good communication and working relationships with the patient, carer and other healthcare professionals were important for successful ACB reduction. Assigning responsibility for prescribing decisions to one named person was suggested to be of high importance. Despite identification of these barriers and facilitators, the limited evidence available results in many remaining uncertainties regarding key areas relevant to the implementation of ACB reduction in practice.

Comparison to a review of the broader concept of ‘deprescribing’ [16] reveals many similarities in findings. In the review by Anderson et al. [16] health professionals’ reluctance to engage with the deprescribing of inappropriate medications was an overarching theme. Similar to the present review, a number of reasons were reported to explain this; conflict with patients, carers or other healthcare professionals, concerns about impact on workload, concerns about losing credibility, risks to patient’s health, lack of knowledge and confidence, and poor communication with other healthcare professionals often resulting in low motivation to tackle deprescribing [16]. Conversely, beliefs that deprescribing was beneficial for patients, concerns about risks of continuation, and confidence to deviate from guidelines were all reported to help enable healthcare professional deprescribing [16]. Those with experience of deprescribing and who had undergone deprescribing training demonstrated more positive attitudes towards deprescribing [16].

Unlike Anderson et al. [16] the present review identified little about patient resistance. A review of barriers and facilitators towards deprescribing from the patient’s perspective demonstrated that patients do generally support deprescribing *if* they understand that it is appropriate for them [18]. Recent surveys suggest that over three quarters of patients and carers are willing to stop at least one medication if a healthcare professional said that it was the best course of action [37, 38]. Yet, health professionals perceive patient willingness to reduce or stop medications as low [39, 40]. One explanation for this mismatch may be the lack of patient involvement in medication reviews [40]. Despite believing that patients should be involved in medication reviews, GPs and pharmacists reported that it was too resource heavy to do so [40]. Concerns that deprescribing reduced health professionals’ credibility have also been raised [16]. However, this was not substantiated in a recent patient-centred deprescribing intervention [41]. Perhaps lack of engagement of patients in the process explains any perceived or realised resistance; it is not the change that patients may resist, but the lack of their involvement.

Conflict arising from collaborations with other professionals, and the system and organisational barriers, are commonly reported in the deprescribing literature [16, 39, 40]. It is evident that a clear process, which fits with existing practices, with shared acceptance and understanding, is important. It remains unclear how this is best achieved. One study proposes a 5-step patient-centred model [42], however the model lacks detail as to how it would be implemented in clinical practice. Only when the challenges are clearly delineated and working solutions found and implemented, can such a model be adopted into routine care.

ACB reduction as a research field is in early development. This may explain the limited number of studies identified and data available for synthesis. However, interventions in this area show promise [15]. Further research into the complex nature of ACB reduction, along with important issues surrounding implementation, will allow these benefits to be realised in clinical care, which can benefit the health and well-being of a number of patients. Several critical research questions have been identified by this review to provide a starting point for future research in this newly emerging field tackling ACB. These include:

- Who should be involved and have responsibility for ACB reduction?
- How can inter-professional collaboration be improved?
- How can ACB reduction be integrated with clinical judgement?
- How might ACB reduction be adapted for different health care systems which involve multiple providers?

This review involved a comprehensive search strategy and was conducted in accordance with methodological recommendations. All study processes were conducted by two independent reviewers (e.g. screening, data extraction, analysis, quality assessment) and an appropriate theoretical framework of analysis was applied to enhance understanding of implementation issues. This is in line with MRC recommendations for understanding complex interventions [20]. However, our review is limited by very few studies, the lack of patient or carer perspectives and the quality of included studies. Only two eligible studies were identified, one of which was of poor quality and high risk of bias, with limited qualitative data for analysis. Both studies exclusively focused upon care for older people. One study [35] presented qualitative data which appeared to be collected opportunistically rather than planned qualitative analysis. Reporting of details regarding the methods of collecting and analysing the qualitative data were poor, resulting in this study being considered very low quality. Presentation of results was also limited in this study. As a consequence, our findings are limited in their breadth and depth, and restricted to the perspectives of healthcare professionals, employed in the Australian healthcare system, towards anticholinergic reduction amongst older people.

## Conclusion

Despite limited studies this review identified several important barriers and facilitators to ACB reduction which require further exploration to enhance the implementation of future ACB reduction interventions. The distinct lack of exploration of patient and carer perceptions risks the development of interventions which are not person-centred, impeding successful implementation. This review identifies several important areas for future research which need to be undertaken to enable the development of successful ACB reduction interventions.

## Supplementary Information

Below is the link to the electronic supplementary material.

Supplementary file 1: Barriers and Facilitators to reducing ACB: A qualitative systematic review (DOCX 34 KB)

## Data Availability

No additional unpublished data are available.
