# Supplementary material for: Barriers and facilitators to reducing anticholinergic burden: a qualitative systematic review
Source: Int J Clin Pharm. 2021 Jun 25;43(6):1451–60. doi: 10.1007/s11096-021-01293-4 (PMC8642323; doi:10.1007/s11096-021-01293-4)
Supplement: Supplementary file 1 — Barriers and Facilitators to reducing ACB: A qualitative systematic review (DOCX 34 KB) [file 11096_2021_1293_MOESM1_ESM.docx]

**Barriers and Facilitators to reducing ACB: A qualitative systematic review**

**Supplementary Information (S1)**

**Contents**

[**Protocol/ Prospero Registration** 2](#_Toc14959093)

[**Database Search Strategies** 7](#_Toc14959094)

[**PRISMA Checklist 2009** 13](#_Toc14959095)

[**ACB Qualitative Barriers & Facilitators Review Coding Sheet** 16](#_Toc14959096)

# **Protocol/ Prospero Registration**

PROSPERO International prospective register of systematic reviews

*Title:* Barriers and facilitators to reducing anticholinergic burden from the perspectives of patients, their carers and healthcare professionals: a qualitative systematic review

*Authors:* Carrie Stewart, Athagran Nakham, Moira Cruickshank, Rumana Newlands, Christine Bond, Phyo Kyaw Myint, Frances Mair, Debi Bhattacharya, Katie Gallacher

*Citation:* Carrie Stewart, Athagran Nakham, Moira Cruickshank, Rumana Newlands, Christine Bond, Phyo Kyaw Myint, Frances Mair, Debi Bhattacharya, Katie Gallacher. Barriers and facilitators to reducing anticholinergic burden from the perspectives of patients, their carers and healthcare professionals: a qualitative systematic review. PROSPERO 2018 CRD42018109084 Available from: http://www.crd.york.ac.uk/PROSPERO/display_record.php?ID=CRD42018109084

*Review question*

What are the barriers and facilitators to reducing patient anticholinergic burden from the perspective of healthcare professionals?

What are the barriers and facilitators to reducing patient anticholinergic burden from the perspective of patients and carers?

*Searches*

Original articles will be searched for within electronic databases including Ovid MEDLINE, EMBASE, CINAHL and PsycINFO. The search strategy will be developed for Ovid MEDLINE and will be adapted for use in other databases where appropriate.

The search strategy will be a combination of exploding MeSH terms (exp.), keywords (kw.) and textwords (tw.).

The strategy will combine terms for population (e.g. Cholinergic Antagonists OR Cholinergic Receptor OR Cholinergic Agents) AND terms for exposure/ intervention (e.g. Inappropriate Prescribing OR Potentially Inappropriate Medication List OR Polypharmacy) AND terms for qualitative research (e.g. Qualitative Research OR Focus Groups OR Interviews).

Searches will be restricted to "human" only studies and no restrictions on publication date. Only studies published in English will be included in this review.

*Types of study to be included*

Only qualitative studies presenting original findings will be included in this review. Systematic reviews and quantitative studies without a qualitative component exploring barriers and faclitators to anticholinergic reduction will be excluded.

*Condition or domain being studied*

Anticholinergic drugs block the actions of acetylcholine and consequentially prevent parasympathetic nerve activity. They may be prescribed or purchased over the counter to treat a range of conditions including gastrointestinal disorders, overactive bladder, allergies, depression. However, anticholinergic drugs have numerous side effects such as dry mouth, constipation, increased heart rate, confusion, and increased risk of falls. The potential impact of anticholinergics on an individual is termed “anticholinergic burden”, reflecting the cumulative effect of concomitant use of multiple medications with anticholinergic properties. Concerns have been raised in relation to persons who may be using such medications, particularly those using multiple anticholinergic drugs. This has resulted in attention towards identifying those at risk and developing interventions to reduce this risk.

*Aim*

Our aim is to systematically review the qualitative literature to identify and explore factors which facilitate or inhibit the implementation of interventions to reduce the anticholinergic burden amongst adult patients.

*Participants/population*

We will include all studies involving adults who are use one or more anticholinergic medication, or carers/ proxy respondents for such adults, or healthcare professionals involved in care of these adults.

We will exclude studies involving persons under 18 years of age or persons not using any anticholinergic medications.

*Intervention(s), exposure(s)*

Any qualitative study which explores barriers and facilitators towards reducing anticholinergic burden from the perspective of patients/ carers or healthcare professionals.

*Comparator(s)/control*

Not applicable.

*Context*

Studies conducted in the following settings will be included:

- Primary care
- Community
- Nursing home
- Outpatient clinics
- Day hospitals/ centres/care facilities
- Rehabilitation services

Studies conducted in the following settings will be excluded:

- Acute/ inpatient care
- Palliative care.

*Main outcome(s)*

Factors which prevent/ inhibit reduction of anticholinergic burden from the prescribers/ healthcare professionals perspective (e.g. pressure from patients).

Factors which support/ promote/ facilitate reduction of anticholinergic burden from the prescribers/ healthcare professionals perspective (e.g. prescribing guidelines).

Factors which prevent/ inhibit reduction of anticholinergic burden from the patients' or carers' perspective (e.g. concerns about efficacy of new medication).

Factors which support/ promote/ facilitate reduction of anticholinergic burden from the patients' or carers' perspective (e.g. understanding of anticholinergic effects).

*Timing and effect measures*

Not applicable

*Additional outcome(s)* None.

Timing and effect measures

*Data extraction (selection and coding)*

Studies identified through database searches will be entered into RefWorks for bibliographic management where duplicates will be removed. They will then be transferred to Covidence for screening. Two reviewers will independently screen titles and abstracts of records to determine whether they potentially meet the inclusion criteria. Full-texts of potentially eligible studies will then be obtained and further examined by two reviewers against the inclusion criteria to determine eligibility. Any disagreements will be resolved by a third independent reviewer from within the research team. Study authors will be contacted where full-texts cannot be sourced. A standardised data extraction form will be used. This will include items from standard reporting and quality assessment checklists, including CASP, Cochrane, and ENTREQ.

*Risk of bias (quality) assessment*

The quality of included studies will be assessed by the CASP checklist for qualitative studies.

*Strategy for data synthesis*

Normalization Process Theory (NPT) will be used to explore, understand and explain qualitative data in relation to factors that act as barriers or facilitators to ACB reduction. NPT is a well-developed theory for understanding such factors from the perspective of intervention implementation. NPT explores how the work of enacting an ensemble of tasks or practices is accomplished through four mechanisms: coherence (sensemaking), cognitive participation (relationship work), collective action (enacting work), and reflexive monitoring (appraisal work).

Data will be analysed by framework synthesis, using a framework underpinned by NPT. A coding sheet detailing and describing the themes required to be explored by NPT will be developed and agreed by the team. This will provide a framework for sorting and categorising the data, and exploring relationships between themes. The framework will be flexible to enable data that falls outside of our conceptual model to be captured.

*Analysis of subgroups or subsets*

None.

*Contact details for further information*

Dr. Carrie Stewart carrie.stewart@abdn.ac.uk

*Organisational affiliation of the review*

University of Aberdeen https://www.abdn.ac.uk/iahs/research/acer/index.php

*Review team members and their organisational affiliations*

- Dr Carrie Stewart. University of Aberdeen
- Mr Athagran Nakham. Univeristy of Aberdeen
- Dr Moira Cruickshank. University of Aberdeen
- Dr Rumana Newlands. University of Aberdeen Professor
- Christine Bond. University of Aberdeen Professor
- Phyo Kyaw Myint. University of Aberdeen
- Professor Frances Mair. University of Glasgow
- Dr Debi Bhattacharya. University of East Anglia
- Dr Katie Gallacher. University of Glasgow

*Type and method of review*

Synthesis of qualitative studies, Systematic review

*Anticipated or actual start date*

20 September 2018

*Anticipated completion date*

31 December 2019

*Funding sources/sponsors*

This study is funded by Dunhill Medical Trust and sponsored by the Institute of Applied Health Sciences, University of Aberdeen.

Conflicts of interest

*None*

Language

*English*

*Country*

England, Scotland

*Published protocol*

*Stage of review*

Review Ongoing

*Subject index terms status*

Subject indexing assigned by CRD

*Subject index terms*

Caregivers; Cholinergic Antagonists; Health Personnel; Humans

*Date of registration in PROSPERO*

12 December 2018

*Date of publication of this version*

12 December 2018

*Details of any existing review of the same topic by the same authors*

*Stage of review at time of this submission*

Stage Started Completed

*Preliminary searches* Yes

*Piloting of the study selection process* Yes

*Formal screening of search results against eligibility criteria* No

*Data extraction*  No

*Risk of bias (quality) assessment* No

*Data analysis*  No

PROSPERO International prospective register of systematic reviews

Versions 12 December 2018

PROSPERO This information has been provided by the named contact for this review. CRD has accepted this information in good faith and registered the review in PROSPERO. The registrant confirms that the information supplied for this submission is accurate and complete. CRD bears no responsibility or liability for the content of this registration record, any associated files or external websites.

Powered by TCPDF (www.tcpdf.org)

# **Database Search Strategies**

**Medline (Ovid)**

| **#** | **Term(s)** |
| --- | --- |
| 1 | exp Cholinergic Antagonists/ |
| 2 | exp Anticholinergic Syndrome/ |
| 3 | exp "Hypnotics and Sedatives"/ |
| 4 | exp Anti-Anxiety Agents/ |
| 5 | exp Antipsychotic Agents/ |
| 6 | exp PSYCHOTROPIC DRUGS/ |
| 7 | exp Histamine Agents/ |
| 8 | exp PARASYMPATHOLYTICS/ |
| 9 | exp Antihypertensive Agents/ |
| 10 | exp ANTICONVULSANTS/ |
| 11 | exp ANTICOAGULANTS/ |
| 12 | exp Urinary Incontinence/ |
| 13 | exp Accidental Falls/ |
| 14 | exp Neurocognitive Disorders/ |
| 15 | exp PARKINSON DISEASE/ |
| 16 | exp PAIN/ |
| 17 | exp DEPRESSION/ |
| 18 | exp ANXIETY/ |
| 19 | exp Vascular Diseases/ |
| 20 | 1 or 2 or 3 or 4 or 5 or 6 or 7 or 8 or 9 or 10 or 11 or 12 or 13 or 14 or 15 or 16 or 17 or 18 or 19 |
| 21 | exp Potentially Inappropriate Medication List/ |
| 22 | exp Inappropriate Prescribing/ |
| 23 | exp POLYPHARMACY/ |
| 24 | exp Deprescriptions/ |
| 25 | (deprescri* or STOPP START or BEERS or FORTA or MAI or Drug Burden Index or Anticholinergic burden or anticholinergic risk or anticholinergic drug or anticholinergic activity or anticholinergic impregnation or anticholinergic scale or anticholinergic exposure or anticholinergic loading or anticholinergic activity).kw. or (deprescrib* or STOPP START or BEERS or FORTA or MAI or Drug Burden Index or Anticholinergic burden or anticholinergic risk or anticholinergic drug or anticholinergic activity or anticholinergic impregnation or anticholinergic scale or anticholinergic exposure or anticholinergic loading or anticholinergic activity).tw. |
| 26 | 21 or 22 or 23 or 24 or 25 |
| 27 | exp Qualitative Research/ |
| 28 | exp Focus Groups/ |
| 29 | exp Interviews as Topic/ |
| 30 | (Qualitative or interview or focus group or ethnograph* or phenomen* or observation*).tw. or (qualitative or interview or focus group or ethnograph* or phenomen* or observation*).kw. |
| 31 | 27 or 28 or 29 or 30 |
| 32 | 20 and 26 and 31 |
| 34 | limit 32 to humans |

**Embase**

| **#** | **Term(s)** |
| --- | --- |
| 1 | exp cholinergic receptor blocking agent/ |
| 2 | exp cholinergic receptor blocking/ |
| 3 | exp cholinergic receptor/ |
| 4 | exp deprescription/ or exp polypharmacy/ |
| 5 | exp inappropriate prescribing/ |
| 6 | exp potentially inappropriate medication/ |
| 7 | exp hypnotic sedative agent/ |
| 8 | exp anxiolytic agent/ |
| 9 | exp psychotropic agent/ |
| 10 | exp neuroleptic agent/ |
| 11 | exp histamine agonist/ |
| 12 | exp antihypertensive agent/ |
| 13 | exp anticonvulsive agent/ |
| 14 | exp anticoagulant agent/ |
| 15 | exp urine incontinence/ |
| 16 | exp falling/ |
| 17 | exp "disorders of higher cerebral function"/ |
| 18 | exp Parkinson disease/ |
| 19 | exp pain/ |
| 20 | exp depression/ |
| 21 | exp anxiety/ |
| 22 | exp vascular disease/ |
| 23 | exp qualitative research/ |
| 24 | exp interview/ |
| 25 | 1 or 2 or 3 or 7 or 8 or 9 or 10 or 11 or 12 or 13 or 14 or 15 or 16 or 17 or 18 or 19 or 20 or 21 or 22 |
| 26 | (deprescri* or STOPP START or BEERS or FORTA or MAI or Drug Burden Index or Anticholinergic burden or anticholinergic risk or anticholinergic drug or anticholinergic activity or anticholinergic impregnation or anticholinergic scale or anticholinergic exposure or anticholinergic loading anticholinergic activity).kw. or (deprescri* or STOPP START or BEERS or FORTA or MAI or Drug Burden Index or Anticholinergic burden or anticholinergic risk or anticholinergic drug or anticholinergic activity or anticholinergic impregnation or anticholinergic scale or anticholinergic exposure or anticholinergic loading anticholinergic activity).tw. |
| 27 | 4 or 5 or 6 or 26 |
| 28 | 25 and 27 |
| 29 | (Qualitative or focus group or interview or ethnograph* or phenomen* or observation*).kw. or (Qualitative or focus group or interview or ethnograph* or phenomen* or observation*).tw. |
| 35 | 23 or 24 or 29 |
| 36 | 28 and 35 |
| 37 | limit 36 to human |

**PsycInfo**

| **#** | **Term(s)** |
| --- | --- |
| 1 | exp cholinergic drugs |
| 2 | exp cholinergic blocking drugs |
| 3 | exp sedatives |
| 4 | exp hypnotic drugs |
| 5 | exp tranquilizing drugs |
| 6 | exp anticonvulsive drugs |
| 7 | exp antihypertensive drugs |
| 8 | exp neuropharmacology |
| 9 | exp anticoagulant drugs |
| 10 | exp antispasmodic drugs |
| 11 | exp histamine |
| 12 | exp urinary incontinence |
| 13 | exp falls |
| 14 | exp Alzheimer’s disease |
| 15 | exp nervous system disorders |
| 16 | exp dementia |
| 17 | exp Parkinson’s disease |
| 18 | exp pain |
| 19 | exp anxiety |
| 20 | exp major depression |
| 21 | exp cerebrovascular disorders |
| 22 | exp cardiovascular disorders |
| 23 | exp polypharmacy |
| 24 | exp drug interactions |
| 25 | exp prescribing (drugs) |
| 26 | (STOPP START OR BEERS or FORTA or MAI or Drug Burden Index or Anticholinergic burden or anticholinergic risk or anticholinergic drug or anticholinergic activity or anticholinergic impregnation or anticholinergic scale or anticholinergic exposure or anticholinergic loading or anticholinergic activity) id. OR (STOPP START OR BEERS or FORTA or MAI or Drug Burden Index or Anticholinergic burden or anticholinergic risk or anticholinergic drug or anticholinergic activity or anticholinergic impregnation or anticholinergic scale or anticholinergic exposure or anticholinergic loading or anticholinergic activity).kw. |
| 27 | despreci*.i.d OR deprescri*.tw. |
| 28 | Exp. qualitative research |
| 29 | (qualitative OR interview OR focus group OR ethnograph* OR phenomen* OR observation*)id. OR (qualitative OR interview OR focus group OR ethnograph* OR phenomen* OR observation*).kw. |
| 30 | 1 or 2 or 3 or 4 or 5 or 6 or 7 or 8 or 9 or 10 or 11 or 12 or 13 or 14 or 15 or 16 or 17 or 18 or 19 or 20 or 21 or 22 |
| 31 | 23 or 24 or 25 or 26 or 27 |
| 32 | 28 or 29 |
| 33 | 30 and 31 and 32 |
| 34 | Limit 33 to human |

**CINAHL**

| **#** | **Term(s)** |
| --- | --- |
| 1 | (MH "Cholinergic Agents+") OR (MH "Cholinergic Agonists+") OR (MH "Cholinergic Antagonists+") |
| 2 | (MM "Polypharmacy") |
| 3 | (MM "Inappropriate Prescribing") |
| 4 | (MH "Hypnotics and Sedatives+") |
| 5 | (MH "Antianxiety Agents+") |
| 6 | (MH "Antipsychotic Agents+") |
| 7 | (MH "Psychotropic Drugs+") |
| 8 | (MH "Histamine Agents+") |
| 9 | (MH "Parasympatholytics+") |
| 10 | (MH "Anticonvulsants+") |
| 11 | (MH "Anticoagulants+") |
| 12 | (MH "Cardiovascular Agents+") |
| 13 | (MH "Incontinence+") |
| 14 | (MM "Accidental Falls") |
| 15 | (MH "Delirium, Dementia, Amnestic, Cognitive Disorders+") |
| 16 | (MM "Parkinson Disease") |
| 17 | (MH "Pain+") |
| 18 | (MH "Depression+") |
| 19 | (MH "Anxiety+") |
| 20 | (MH "Vascular Diseases+") |
| 21 | (MM "Focus Groups") |
| 22 | (MM "Semi-Structured Interview") |
| 23 | (MH "Qualitative Studies+") |
| 24 | TX STOPP START OR BEERS OR FORTA OR MAI OR Drug Burden Index or Anticholinergic burden or anticholinergic risk or anticholinergic drug or anticholinergic activity or anticholinergic impregnation or anticholinergic scale or anticholinergic exposure or anticholinergic loading or anticholinergic activity |
| 25 | TX deprescri* |
| 26 | TX qualitative OR interview OR focus group OR ethnogra* OR phenomen* OR observation* |
| 27 | 1 OR 4 OR 5 OR 6 OR 7 OR 8 OR 9 OR 10 OR 11 OR 12 OR 13 OR 14 OR 15 OR 16 OR 17 OR 18 OR 19 OR 20 |
| 28 | 2 OR 3 OR 24 OR 25 |
| 29 | 21 OR 22 OR 23 OR 26 |
| 30 | 27 AND 28 AND 29 |
| 31 | Limit 30 to human |

# **PRISMA Checklist 2009**

| **Section/topic** | **#** | **Checklist item** | **Reported on page #** |
| --- | --- | --- | --- |
| **TITLE** | | |  |
| Title | 1 | Identify the report as a systematic review, meta-analysis, or both. | 1 |
| **ABSTRACT** | | |  |
| Structured summary | 2 | Provide a structured summary including, as applicable: background; objectives; data sources; study eligibility criteria, participants, and interventions; study appraisal and synthesis methods; results; limitations; conclusions and implications of key findings; systematic review registration number. | 1 |
| **INTRODUCTION** | | |  |
| Rationale | 3 | Describe the rationale for the review in the context of what is already known. | 3 |
| Objectives | 4 | Provide an explicit statement of questions being addressed with reference to participants, interventions, comparisons, outcomes, and study design (PICOS). | 3, 14 |
| **METHODS** | | |  |
| Protocol and registration | 5 | Indicate if a review protocol exists, if and where it can be accessed (e.g., Web address), and, if available, provide registration information including registration number. | 3 |
| Eligibility criteria | 6 | Specify study characteristics (e.g., PICOS, length of follow-up) and report characteristics (e.g., years considered, language, publication status) used as criteria for eligibility, giving rationale. | 4, 14 |
| Information sources | 7 | Describe all information sources (e.g., databases with dates of coverage, contact with study authors to identify additional studies) in the search and date last searched. | 4, S1 |
| Search | 8 | Present full electronic search strategy for at least one database, including any limits used, such that it could be repeated. | S1 |
| Study selection | 9 | State the process for selecting studies (i.e., screening, eligibility, included in systematic review, and, if applicable, included in the meta-analysis). | 4 |
| Data collection process | 10 | Describe method of data extraction from reports (e.g., piloted forms, independently, in duplicate) and any processes for obtaining and confirming data from investigators. | 4 |
| Data items | 11 | List and define all variables for which data were sought (e.g., PICOS, funding sources) and any assumptions and simplifications made. | 4 |
| Risk of bias in individual studies | 12 | Describe methods used for assessing risk of bias of individual studies (including specification of whether this was done at the study or outcome level), and how this information is to be used in any data synthesis. | 4 |
| Summary measures | 13 | State the principal summary measures (e.g., risk ratio, difference in means). | NA |
| Synthesis of results | 14 | Describe the methods of handling data and combining results of studies, if done, including measures of consistency (e.g., I^2^) for each meta-analysis. | 4 |

| **Section/topic** | **#** | **Checklist item** | **Reported on page #** |
| --- | --- | --- | --- |
| Risk of bias across studies | 15 | Specify any assessment of risk of bias that may affect the cumulative evidence (e.g., publication bias, selective reporting within studies). | 4 |
| Additional analyses | 16 | Describe methods of additional analyses (e.g., sensitivity or subgroup analyses, meta-regression), if done, indicating which were pre-specified. | NA |
| **RESULTS** | | |  |
| Study selection | 17 | Give numbers of studies screened, assessed for eligibility, and included in the review, with reasons for exclusions at each stage, ideally with a flow diagram. | 5 |
| Study characteristics | 18 | For each study, present characteristics for which data were extracted (e.g., study size, PICOS, follow-up period) and provide the citations. | 5 |
| Risk of bias within studies | 19 | Present data on risk of bias of each study and, if available, any outcome level assessment (see item 12). | 15 |
| Results of individual studies | 20 | For all outcomes considered (benefits or harms), present, for each study: (a) simple summary data for each intervention group (b) effect estimates and confidence intervals, ideally with a forest plot. | NA |
| Synthesis of results | 21 | Present results of each meta-analysis done, including confidence intervals and measures of consistency. | NA |
| Risk of bias across studies | 22 | Present results of any assessment of risk of bias across studies (see Item 15). | 15 |
| Additional analysis | 23 | Give results of additional analyses, if done (e.g., sensitivity or subgroup analyses, meta-regression [see Item 16]). | NA |
| **DISCUSSION** | | |  |
| Summary of evidence | 24 | Summarize the main findings including the strength of evidence for each main outcome; consider their relevance to key groups (e.g., healthcare providers, users, and policy makers). | 10 |
| Limitations | 25 | Discuss limitations at study and outcome level (e.g., risk of bias), and at review-level (e.g., incomplete retrieval of identified research, reporting bias). | 10 |
| Conclusions | 26 | Provide a general interpretation of the results in the context of other evidence, and implications for future research. | 10-11 |
| **FUNDING** | | |  |
| Funding | 27 | Describe sources of funding for the systematic review and other support (e.g., supply of data); role of funders for the systematic review. | 11 |

*From:*  Moher D, Liberati A, Tetzlaff J, Altman DG, The PRISMA Group (2009). Preferred Reporting Items for Systematic Reviews and Meta-Analyses: The PRISMA Statement. PLoS Med 6(7): e1000097. doi:10.1371/journal.pmed1000097

For more information, visit: **www.prisma-statement.org**.

# **ACB Qualitative Barriers & Facilitators Review Coding Sheet**

| **1.Coherence (Sense making)** | |
| --- | --- |
| 1.1 Differentiation | Is there a clear understanding of how a specific ACB reduction intervention would differ from existing practice? |
| 1.2 Communal specification | Do individuals have a shared understanding of the aims, objectives and expected benefits of an ACB reduction intervention? |
| 1.3 Individual specification | Do individuals have a clear understanding of their specific tasks and responsibilities in the implementation of an ACB reduction intervention? |
| 1.4 Internalization | Do individuals understand the value, benefits and importance of the ACB reduction intervention? |
| 1.5 Coherence (other) |  |
| **2. Cognitive Participation (Relationship work)** | |
| 2.1 Enrolment | Do individuals buy into the idea of the ACB reduction intervention? |
| 2.2 Activation | Can individuals sustain involvement? |
| 2.3 Initiation | Are key individuals willing to drive the implementation? |
| 2.4 Legitimation | Do individuals believe it is right for them to be involved? |
| 2.5 Cognitive Participation (other) |  |
| **3. Collective Action (Enacting work)** | |
| 3.1 Skill set workability | How does innovation affect roles and responsibilities or training needs? |
| 3.2 Contextual Integration | Is there organisational support? |
| 3.3 Interactional workability | Does the ACB reduction intervention make peoples work easier? |
| 3.4 Relational integration | Do individuals have confidence in ACB reduction intervention? |
| 3.5 Collective action (other) |  |
| **4. Reflexive Monitoring (Appraisal work)** | |
| 4.1 Reconfiguration | Do individuals try to alter the ACB reduction intervention? |
| 4.2 Communal appraisal | How do groups judge the value of the ACB reduction intervention? |
| 4.3 Individual appraisal | How do individuals appraise the effects on them and their work environment? |
| 4.4 Systematization | How are the benefits or problems identified or measured? |
| 4.5 Reflexive Monitoring (other) |  |
| **5. Other** | |
| 5.1 Other | Anything felt relevant but not covered by any of the previous codes |
